# Supplementary material for: Vacuum laser acceleration of super-ponderomotive electrons using relativistic transparency injection
Source: Nat Commun. 2022 Jan 10;13:54. doi: 10.1038/s41467-021-27691-w (PMC8749006; doi:10.1038/s41467-021-27691-w)
Supplement: Supplementary file 1 — Supplementary Information [file 41467_2021_27691_MOESM1_ESM.pdf]

Supplementary Information for "Vacuum laser acceleration of super-ponderomotive electrons using relativistic transparency injection"

P. K. Singh[1, †], F.-Y. Li[1, †], C.-K. Huang[1], A. Moreau[2], R. Hollinger[2], A. Junghans[1], A. Favalli[1], C. Calvi[3], S. Wang[2], Y. Wang[2], H. Song[2], J. J. Rocca[2,3], B. Reinovsky[1], and S. Palaniyappan[1]*

[1]Los Alamos National Laboratory, Los Alamos, 87545, New Mexico, USA.

[2]Department of Electrical and Computer Engineering, Colorado State University, Fort Collins, CO, 80523, USA.

[3]Department of Physics, Colorado State University, Fort Collins, Colorado, 80523, USA.

[†]These authors contributed equally.

[*]Corresponding author: sasi@lanl.gov

Supplementary Note 1

Characterization of laser focal spot and intensity. The focal profile of the laser pulse, focused by a F/2 off-axis parabolic dielectric mirror was measured with 50× magnification objective system, showing a spot size of 1.7μm×1.81μm (FWHM) which had 32% concentration of the total laser energy. During the experiment, the frequency-doubled (400 nm) laser, of 75 fs (FWHM) pulse duration, and 6.8 J maximum energy (for full pulse duration and full spot), was focused on the target surface. The resultant estimated peak intensity reaches $0.9 \times 10^{21}\, Wcm^{-2}$. Supplementary Fig. 1 shows the laser focal intensity distribution, both in linear and log scale. In estimating the peak intensity, we used a rectangle spot of area 1.7μm×1.81μm, instead of an ellipse, due to the limited number of pixels encircled by the spot in the CCD image.

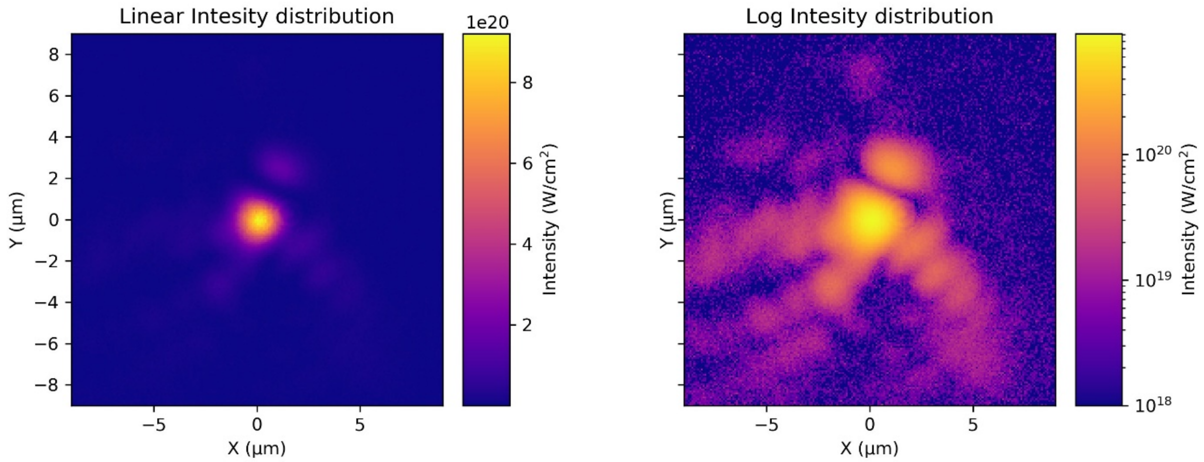

Supplementary Figure 1. Characterization of laser focal spot and intensity. Estimated laser focal intensity in linear (left panel) and in log (right panel) scale. The distribution is obtained for a low-energy shot and scaled to the maximum 6.8J energy. The intensity profile at the full laser amplification may differ from the one using only the pilot.

Supplementary Note 2

Complete data of back-reflected laser spectrum. Supplementary Fig. 2 summarizes the back-reflected spectra obtained for a wide range of foil thickness from partially transparent 5 nm to completely opaque 200 nm; see the Methods for the detailed setup of the measurement. It is seen that the spectra are nearly identical for all thicknesses (Supplementary Fig. 2a) and show a predominant spectral broadening of 50 – 60 nm and a redshift of nearly 10 nm relative to the input laser spectrum (Supplementary Fig. 2b). For our experimental conditions, the spectral broadening could be introduced via relativistic mass effect. The instantaneous frequency shift is given as $\Delta\omega = (\omega_{inst} - \omega_0) = -\delta\phi/\delta t = -\frac{\omega_0}{c}\int \frac{\delta\eta}{\delta t}\, dx$, where the relativistic corrected plasma refractive index ($\eta = \sqrt{(1 - n_e/\gamma n_c)}$), n_e, is the electron density, n_c is the plasma critical density, $\gamma = \sqrt{1 + a_0^2/2}$ is average Lorentz factor of electron and a_0 is laser strength parameter. At the rising part of the laser pulse, the γ increase which causes increase of the plasma refractive index ($\eta = \sqrt{(1 - n_e/\gamma n_c)}$), and therefore introduces a red shift ($\frac{\delta\gamma}{\delta t} > 0$; $\frac{\delta\eta}{\delta t} > 0$; $\Delta\omega < 0$). After passing the peak of the laser pulse, the γ decrease which causes decrease of the plasma refractive index and therefore introduces a blue shift ($\frac{\delta\gamma}{\delta t} < 0$; $\frac{\delta\eta}{\delta t} < 0$; $\Delta\omega > 0$). Since our laser pulse is symmetric in time, we are observing quite symmetric blue and red spectral broadening. The observation of back-reflected spectrum being similar for all the targets, regardless of thickness, as a coarse indicator, could imply that the laser contrast is good enough to prevent any prepulse induced target deterioration, especially for the ultrathin, few nanometer foils. In presence of strong prepulse, the thin targets could be more susceptible to prepulse driven foil expansion, as there is only finite mass available.

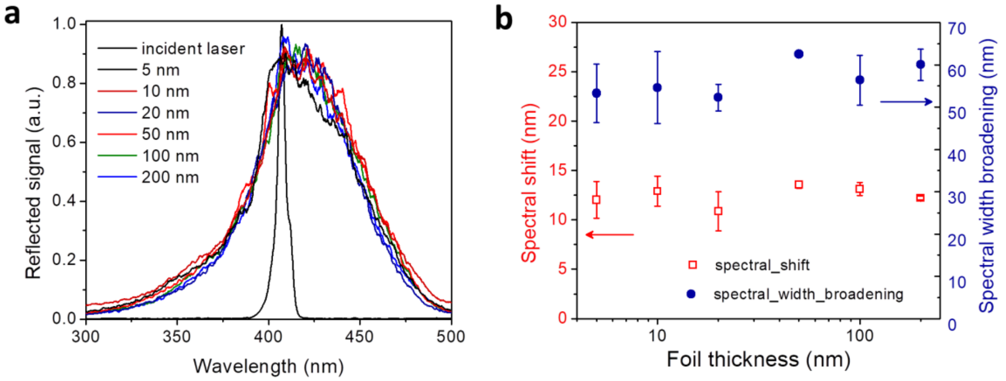

Supplementary Figure 2. Complete data of back-reflected laser spectrum. (a) Back-reflected spectra measured for different target thickness ranging from 5 nm to 200 nm. (b) The variation of spectral width and central shift with respect to the incident laser spectrum. The error bars represent the standard deviation of the data from all the corresponding shots.

Supplementary Note 3

Contribution of x-rays to the image-plate data. During the electron spatial beam-profile measurement, the stack of image plates (IPs) is protected by a 220 µm thick Cu foil (Supplementary Fig. 3a) which blocks x-ray photons up to 20 keV, protons up to 50 MeV, and low-energy electrons up to 400 keV. As the maximum proton energy recorded in our experiments is less than 10 MeV, we can safely rule out the contribution of ions to the IP data. However, x-rays of energy more than 20 keV are generated in the experiment, which may influence the IP data. To distinguish the contribution of x-rays from fast electrons, a dipole magnet (0.2 T) is inserted between the target and IPs (Supplementary Fig. 3e) to deflect electrons

and ions to sides, leaving mainly undeflected x-ray photons to reach the IPs. Strongly deflected electrons, bunched on the right side of the IPs, can be seen in Supplementary Figs. 3f-h. Except for the first IP, the x-ray contribution is observed to be at least one order of magnitude weaker than that from fast electrons by comparing with Supplementary Figs. 3b-d.

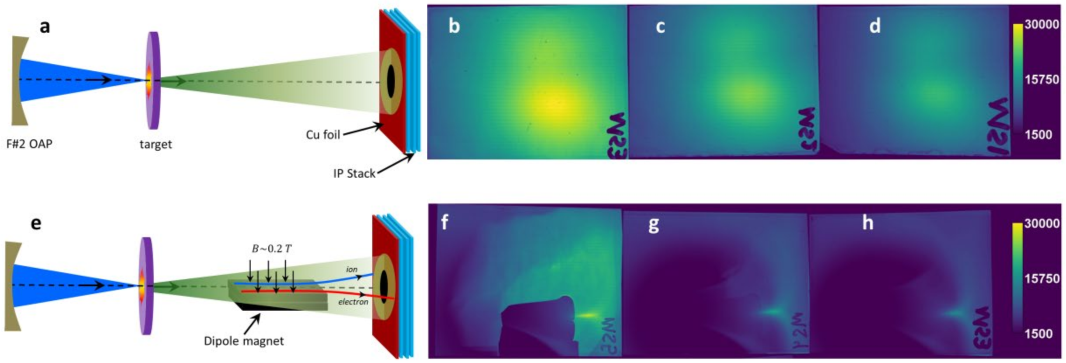

Supplementary Figure 3. Contribution of x-rays to the image-plate data. (a) Sketch of the measurement without a dipole magnet. The right panels (b-d) show corresponding image-plate data recorded for the first three layers. (e-h) The same measurement as the top panels but with a dipole magnet.

Supplementary Note 4

Representativeness of d = 20 nm for characterizing the RT regime in simulations. In the main paper, we have simulated the RT regime using a 20 nm thick foil instead of the nominal 5 nm foil as used in the experiments. This is partly due to the inherent uncertainties in foil thickness for targets thinner than 50 nm, as informed by the target manufacturer. The actual thickness may also increase due to vapor contamination arising from non-ideal vacuum conditions which more affect the thinnest targets. Moreover, due to the uncertainties in the focal spot quality (hence peak intensity), it was challenging to quantitatively reproduce all experimental findings while sticking to the nominal laser-target parameters. More importantly, the objective of our simulations was to identify the key physics that are pertinent to the novel effect of RT irrespective of a specific set of parameters. For these considerations, we have explored closely around the nominal parameters, and here we present (Supplementary Fig. 4) 2D PIC results for a range of target thickness up to 50 nm for the same laser intensity as used in the main text (i.e., Supplementary Fig. 4). The target was found to be completely opaque (i.e., no laser transmission) for $d > 30$ nm. However, as long as finite transmission happens ($d \leq 30$ nm), the electron beam consistently displays a central dip in the transverse profile and much enhanced acceleration. These features are typical for the RT regime as found in experiments. These supplemented simulations thus prove that the presented dynamics with the 20 nm foil is representative for the RT regime.

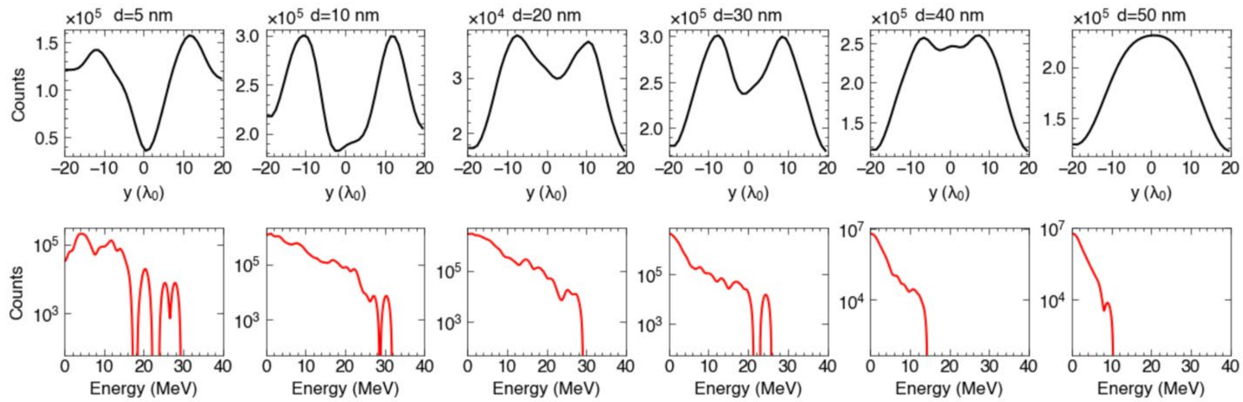

Supplementary Figure 4. **Electron acceleration from 2D PIC simulations for a range of target thickness.** (upper panels) Spatial distribution and (lower panels) energy spectrum of the electrons collected by a virtual detector as described in the Methods. From left to right the target thickness varies from 5 nm to 50 nm. The y axis is adjusted such that y=0 corresponds to the laser central axis.

Supplementary Note 5

3D PIC simulation with a_0=10. The following supplementary 3D PIC simulations (Supplementary Fig. 5) used a_0=10, corresponding to the nominal intensity $\sim 0.9 \times 10^{21} W/cm^2$. These two simulations show the same contrast in the density evolution (a) and energy spectrum (b) between d = 20 nm (RT regime) and 200 nm (opaque regime) as found for a_0=5. In particular, particle tracking result (c) shows the same VLA mechanism where the electrons gain the most energy from acceleration in the rear vacuum space. These additional simulations demonstrate that the mechanism clarified in the main paper applies to a wide range of laser intensities so along as the RT process is involved.

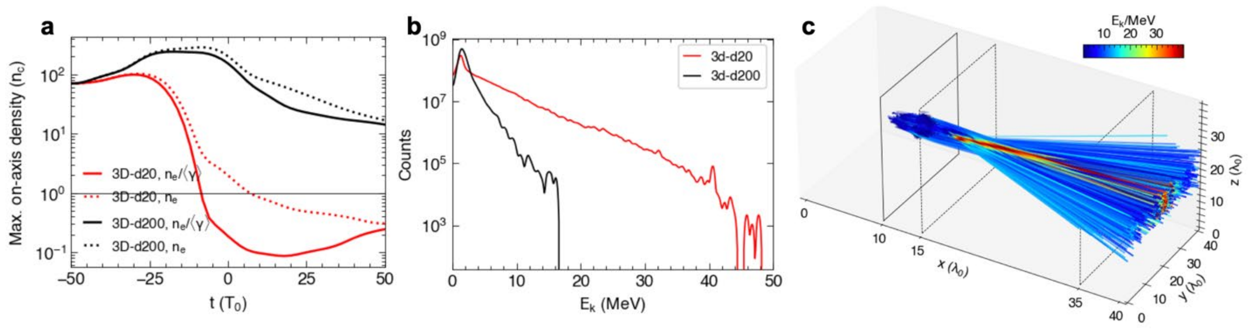

Supplementary Figure 5. Electron acceleration from a 3D PIC simulation with a_0=10. (a) On-axis density (both normal and effective) evolution for both d =20 nm and 200 nm cases. (b) The electron energy spectra collected by a virtual screen placed $25\lambda_0$ behind the foil (at x=$10\lambda_0$) at the end of the simulations (t=$150T_0$). (c) Trajectories (colored by instantaneous energy) of those tracking electrons that acquire a maximum $p_x/m_e c > 60$ during the interaction. The other laser-target parameters are kept the same as used in the main paper.

Supplementary Note 6

Enhanced laser self-focusing and diffraction in 3D PIC simulation of the RT regime. Supplementary Fig. 6 shows the evolution of laser peak amplitude in the 3D simulation of the RT regime and its comparison

with 2D using the same laser parameters. It is seen that the 3D laser shows ~ 40% stronger self-focusing than in 2D during the volumetric interaction (RT sets in at t ~ -5T_0), but also diffracts much faster during t > 10T_0, after it transmits through the focal plane (i.e., initial foil position). The difference in diffraction is partly caused by Gaussian beam optics where a 2D pulse diffracts slower by a factor of $\sqrt{W/W_0}$ compared to 3D; $W = W_0\sqrt{1+(z/z_R)^2}$ is the beam waist at position z, W_0 the waist at $z = 0$, and $z_R = \pi W_0^2 / \lambda_0$ the Rayleigh length. Additionally, the stronger focusing in 3D may result in a tighter laser beam radius and a smaller aperture size in the target due to RT, increasing the diffraction angles. Methods to mitigate these effects, using a larger focal spot for example, would be of interest for optimizing VLA especially in the rear space of the target.

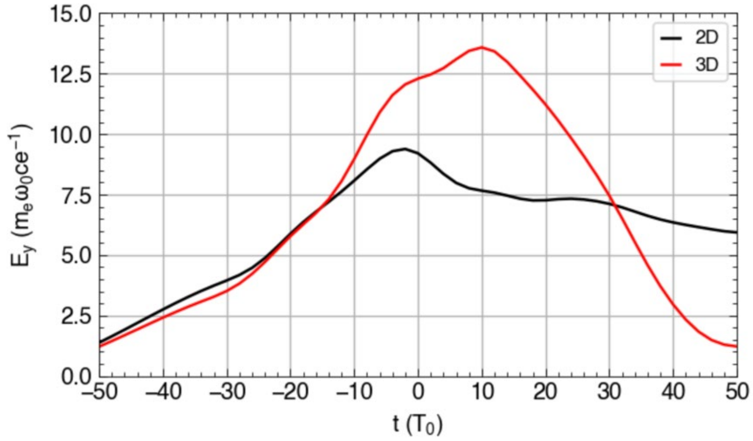

Supplementary Figure 6. Comparison of laser evolution between 2D and 3D PIC simulations. The black curve shows the evolution of laser peak amplitude in 2D simulation. The red curve shows 3D result. The simulations used the 20 nm RT foil. The time axis is adjusted such that t = 0 corresponds to the arrival of the laser peak.
